# Windthrow causes declines in carbohydrate and phenolic concentrations and increased monoterpene emission in Norway spruce

**DOI:** 10.1371/journal.pone.0302714

**Published:** 2024-05-28

**Authors:** Linda M. A. Lehmanski, Lara M. Kösters, Jianbei Huang, Martin Göbel, Jonathan Gershenzon, Henrik Hartmann

**Affiliations:** 1 Department of Biogeochemical Processes, Max Planck Institute for Biogeochemistry, Jena, Germany; 2 Department of Biogeochemical Integration, Max Planck Institute for Biogeochemistry, Jena, Germany; 3 Department for Biochemistry, Max Planck Institute for Chemical Ecology, Jena, Germany; 4 Institute for Forest Protection, Julius Kühn-Institute Federal Research Centre for Cultivated Plants, Quedlinburg, Germany; 5 Faculty of Forest Sciences and Forest Ecology, Georg-August-University Göttingen, Göttingen, Germany; Technical University in Zvolen, SLOVAKIA

## Abstract

With the increasing frequencies of extreme weather events caused by climate change, the risk of forest damage from insect attacks grows. Storms and droughts can damage and weaken trees, reduce tree vigour and defence capacity and thus provide host trees that can be successfully attacked by damaging insects, as often observed in Norway spruce stands attacked by the Eurasian spruce bark beetle *Ips typographus*. Following storms, partially uprooted trees with grounded crowns suffer reduced water uptake and carbon assimilation, which may lower their vigour and decrease their ability to defend against insect attack. We conducted *in situ* measurements on windthrown and standing control trees to determine the concentrations of non-structural carbohydrates (NSCs), of phenolic defences and volatile monoterpene emissions. These are the main storage and defence compounds responsible for beetle´s pioneer success and host tree selection. Our results show that while sugar and phenolic concentrations of standing trees remained rather constant over a 4-month period, windthrown trees experienced a decrease of 78% and 37% of sugar and phenolic concentrations, respectively. This strong decline was especially pronounced for fructose (-83%) and glucose (-85%) and for taxifolin (-50.1%). Windthrown trees emitted 25 times greater monoterpene concentrations than standing trees, in particular alpha-pinene (23 times greater), beta-pinene (27 times greater) and 3-carene (90 times greater). We conclude that windthrown trees exhibited reduced resources of anti-herbivore and anti-pathogen defence compounds needed for the response to herbivore attack. The enhanced emission rates of volatile terpenes from windthrown trees may provide olfactory cues during bark beetle early swarming related to altered tree defences. Our results contribute to the knowledge of fallen trees vigour and their defence capacity during the first months after the wind-throw disturbance. Yet, the influence of different emission rates and profiles on bark beetle behaviour and host selection requires further investigation.

## Introduction

In recent years, forests throughout the world have suffered an increased number of extreme weather events such as storms, droughts and fires caused by climate change that promote large-scale tree mortality [1]. The occurrence and intensity of forest damaging storms has risen, especially in Central Europe, recent examples being the low-pressure systems Ylenia and Zeynep in February 2022 [2, 3]. Storms leading to uprooting of trees (windthrow), have strong effects on forest biodiversity, soil properties, vegetation and micro-climate as well as on insect communities [4, 5]. Drought stress or uprooting caused by storm damage can limit water uptake of trees and therefore have strong effects on tree physiology and vitality of trees [6]. Because photosynthesis is reduced and less non-structural carbohydrates are available for the production of defensive compounds, such as terpenes or phenolics, trees can become more susceptible to successful colonisation by bark-boring insects [7–10]. Phenolics in particular have been shown to play an important role in tree defence against bark beetles and their symbiotic microbes [11]. The recent large-scale die-off events of Norway spruce (*Picea abies*) caused by its main pest the Eurasian Spruce bark beetle *Ips typographus* (L.) (Coleoptera: Scolytidae) are a bold example of how abiotic stress can cause or facilitate insect infestations. Spruce has been planted abundantly in many regions in Germany because trees grow fast and the wood is of high quality for many applications; however, spruce is also particularly predisposed to suffer from drought and windthrow [12]. Wind-felled spruce trees offer ideal breeding material for certain insect pests which can trigger population increases that can also threaten standing, healthy trees [4–6]. While vigorous trees are of higher nutritional value for bark beetles, they are generally well defended against successful beetle attack, at least when beetle population densities are low (endemic) [13]. By contrast, bark beetles seem to be more successful in attacking and colonising trees that were exposed to disturbance and stresses like windthrow or drought [7–10].

In springtime, overwintering pioneer beetles emerge from within the bark or forest litter to colonise trees and establish the first annual brood. Being exhausted from overwintering these beetles thus need to locate suitable hosts that are weak enough to ensure a successful colonisation and egg deposition, while providing sufficient resources to allow beetle development until maturation [14, 15]. Hence, finding the right host is a crucial aspect of the beetle’s life cycle to ensure the survival of the population, a process termed primary attraction that likely is guided by cues emitted from trees, although there currently is no coherent evidence for primary attraction [16]. Several studies have been conducted to address aspects of attraction of beetles to certain cues [17–21], yet results do not clearly show that beetles can differentiate between stressed and unstressed trees. Following first attack, emission profiles substantially change as pioneer beetles and their associated microorganisms start emitting pheromones [22] to attract conspecifics (i.e. secondary attraction) [11].

In this study we investigated the effect of windthrow on tree vitality and defence capacity as well as volatile emissions. Therefore, we examined sugar and phenolic concentrations and volatile emission profiles of stressed (windthrown, weakened) and unstressed (standing, vigorous) trees. We hypothesised that (1) windthrow will lead to decreasing carbohydrate availability in tree stems due to reduced water uptake and light exposure. This will in turn (2) lead to reduced local (i.e. stem) production of secondary metabolites, like phenolics, that are required to defend against attacking bark beetles, and (3) these changes in tree metabolism will be accompanied by quantitative and qualitative changes in emissions of monoterpene volatiles that are proposed to be the main factor influencing early swarming bark beetle behaviour during the search for a suitable host.

## Methods and materials

### Site

In February 2022, successive storms with wind speeds up to 120 km/h caused major damage to forests in Germany [23], producing many windthrown trees as breeding material for resident bark beetles. Measurements were performed in a 50-70-year-old Norway spruce stand in Schöngleina (near Jena, Thuringia, Germany) which was also partially damaged by windthrow. In March, we selected 6 windthrown trees and 6 visually healthy standing trees for our measurements. Measurements were conducted from March ‐ June 2022.

### Stem bark sampling

**Fig 1:**
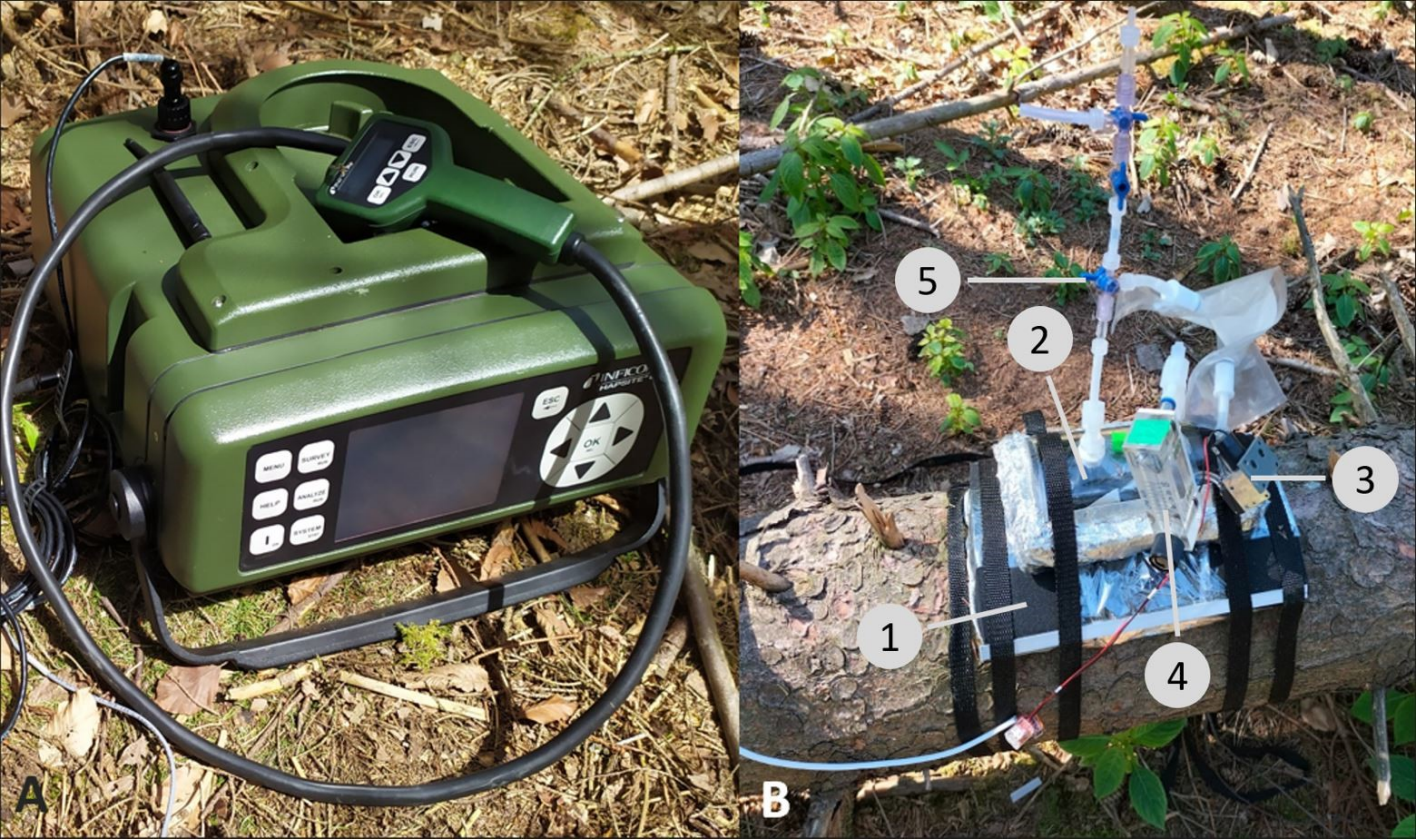
A: Mobile GC-MS (Gas chromatography–mass spectrometry), with attached probe for volatile collections; B: Dynamic volatile collection chamber attached to a windthrown trees. A permanent frame (1) is attached to the stem on which the collection chamber (2) is strapped. A pump (3) and flowmeter (4) allow constant air flow. A valve (5) can be opened for rinsing with ambient air and taking air samples with the mobile GC-MS. All materials are made from VOC repelling materials.

Bark samples were collected at 4 different time points during the experiment. We used an 18 mm hole punch to cut out pieces of bark/phloem on the opposite side of the chamber frames (see Fig 1) to prevent direct effects on tree physiology and therefore on volatile emissions. Samples were put in dry ice for transport to the lab and stored in a -20°C freezer until they were freeze dried once all samples had been collected.

### Sugar measurements

Soluble sugars were analysed as described by [24] in detail. In a nutshell: 10mg of ground bark material was extracted with 0.5 mL of 85% ethanol, then vortexed 1 min, incubated for 10 min at 90°C and centrifuged for 1 min at 13,000g. The supernatant was collected and the extraction step was repeated after which the supernatants were combined, diluted and analysed with a High-Performance Liquid Chromatography coupled to a Pulsed Amperometric Detection (HPLC-PAD) following [25]. To determine the total soluble sugar concentrations, we summed concentrations of glucose, sucrose and fructose.

### Phenolic measurements

Phenolic compounds were extracted as described in Huang et al. 2017 and 2019, with slight modifications [26, 27]. Briefly, ∼30 mg of ground freeze-dried sample was extracted with 1 ml methanol containing 20 μg of apigenin-7-glucoside (Carl Roth GmbH, Germany) as an internal standard. The mixture was vortexed for 10 min and centrifuged for 10 min at 13,000g at room temperature. The supernatant was collected, and the pellet was re-extracted with 0.8 ml methanol containing the internal standard. Both supernatants were combined and analysed using HPLC-mass spectrometry (MS) (HPLC, Agilent, Santa Clara, CA, USA; MS, Sciex, Darmstadt, Germany). Phenolic compounds were separated on a Zorbax Eclipse XDB-C18 column (4.6 x 50 mm, 1.8 μm; Agilent) using mobile phase 0.05% (v/v) formic acid (phase A) and acetonitrile (phase B) at a flow rate 1.1 ml min-1, with the following profile: 0–1 min, 100% A, 0% B; 1–7 min, 0–65% B; 7–7.01 min, 65–100% B; 7.01–8 min, 100% B; 8–8.01 min, 100–0% B; 8.01–10 min, 0% B. The MS was operated as follows: negative ionization mode; ion spray voltage, -4200 V; turbo gas temperature, 700°C; nebulizing gas, 70 p.s.i.; curtain gas, 30 p.s.i.; heating gas, 60 p.s.i.; and collision gas at 10 p.s.i. Multiple reaction monitoring was used to analyse the parent ion → product ion: m/z 288.9 → 109.1 for catechin; m/z 404.8 → 243 for astringin; and m/z 418.9 → 257.1 for isorhapontin. The sum of catechin and proanthocyanidin B1 was reported as flavan-3-ols. The sum of astringin and isorhapontin was reported as stilbenes. Compounds were identified by comparison of retention time and mass spectra with standards and quantified using the peak area in relation to the internal standard peak area. The response factors were calculated with standards [28]. For the determination of total phenolic concentration, all compound concentrations were summed.

### Volatile measurements

Volatile measurements were performed from May to June 2022 using a closed dynamic chamber system fixed to the stem of each tree and attached to a mobile gas chromatograph–mass spectrometer (GC-MS, HAPSITE, Inficon, Switzerland, Fig 1). We attached permanently installed flexible frames made from closed porous cell foam (EPDM, ethylene propylene diene monomer rubber) covered in oven bags (Cofresco Frischhalteprodukte GmbH & Co. KG) to relatively smooth and undamaged bark areas. The chamber system itself consisted of a pump (model NMPO15S, KNF Group, Freiburg, Germany), a controllable flowmeter (model FR2A13BVBN, Key Instruments, Croydon, US) to ensure constant air flow, a chamber (320 mL) which was lined with Polyethylenterephthalat (PET) oven bags to which volatiles do not adhere, and hoses and connecting pieces made from FEP and PFA or stainless steel. All materials were chosen to prevent volatile adherence and thus cross contamination between samples. After installation on the tree, we flushed the chamber for 5 min at a flow rate of 0.4 L min^-1^ with ambient air that was cleaned of volatiles using an active charcoal filter. To increase concentrations of volatiles in the headspace, we incubated the chamber for 30 min with an air flow of 0.4 L/min. The mobile GC-MS then sampled 150 mL of air from the headspace and collected the volatiles on its concentrator column. The mobile GC-MS was pre-programmed following [28]. The starting temperatures were: column, 60°C; membrane, 80°C; valve oven, 70°C; heated lines, 70°C; probe, 40°C. For compound separation, we used a 15 m fused silica Restek Rtx-1 MS capillary GC column (5% diphenyl/95% dimethyl polysiloxane phase,0.25 mm inner diameter, 1 μm film thickness). Desorption of volatiles from the absorbent happened with the following temperatures: 60°C hold for 1 min, followed by an increase to 120 at 30°C min−1, hold for 15 min, and then to 200°C at 30°C min−1, hold for 2 min. MS was done in the electron ionization mode at 70 eV with N2 as carrier gas. For analysis, we used a Hapsite software ER Analysis. We identified compounds by comparing mass spectra with the reference spectra of the NIST library and ensured by the measurement of standards (α-pinene, camphene, β-pinene, 3-carene, limonene (Merck, KGaA). Quantification was done using an internal standard of the mobile GC-MS, Bromopentafluorobenzene.

### Data analysis

We used Shapiro-Wilk and Levene’s tests to examine normal distribution and homogeneity of variances. If normal distribution occurred, we tested differences of treatments with non-paired t-tests. Where normality and homogeneity of variances were not met, we applied non-parametric tests. To test whether means of independent treatments were equal, we performed Mann-Whitney-U tests. Kolmogorov Smirnov tests showed if distributions between compounds and treatments differed.

We normalized the data to account for the large differences in absolute values between treatments and sampling. This was done following the subsequent equation by minimum and maximum concentration for individual compounds for both treatments where Xi is the concentration of a compound on day i.


$$norm(xi)=\frac{xi-\min(\;compound)}{\max(compound)-\min(compound)}$$

Data and statistical analysis was performed using R (version 4.13, R Development Core Team 2023) [29]. Code used for statistical analysis can be found at https://github.com/LLehmanski/windthrow.

The forestry office Jena-Holzland in Stadtroda, Germany granted permission for our field research. Consent from IRB or an ethics committee was not necessary. No field permit number was issued.

## Results

### Sugar and phenolic concentrations

**Fig 2:**
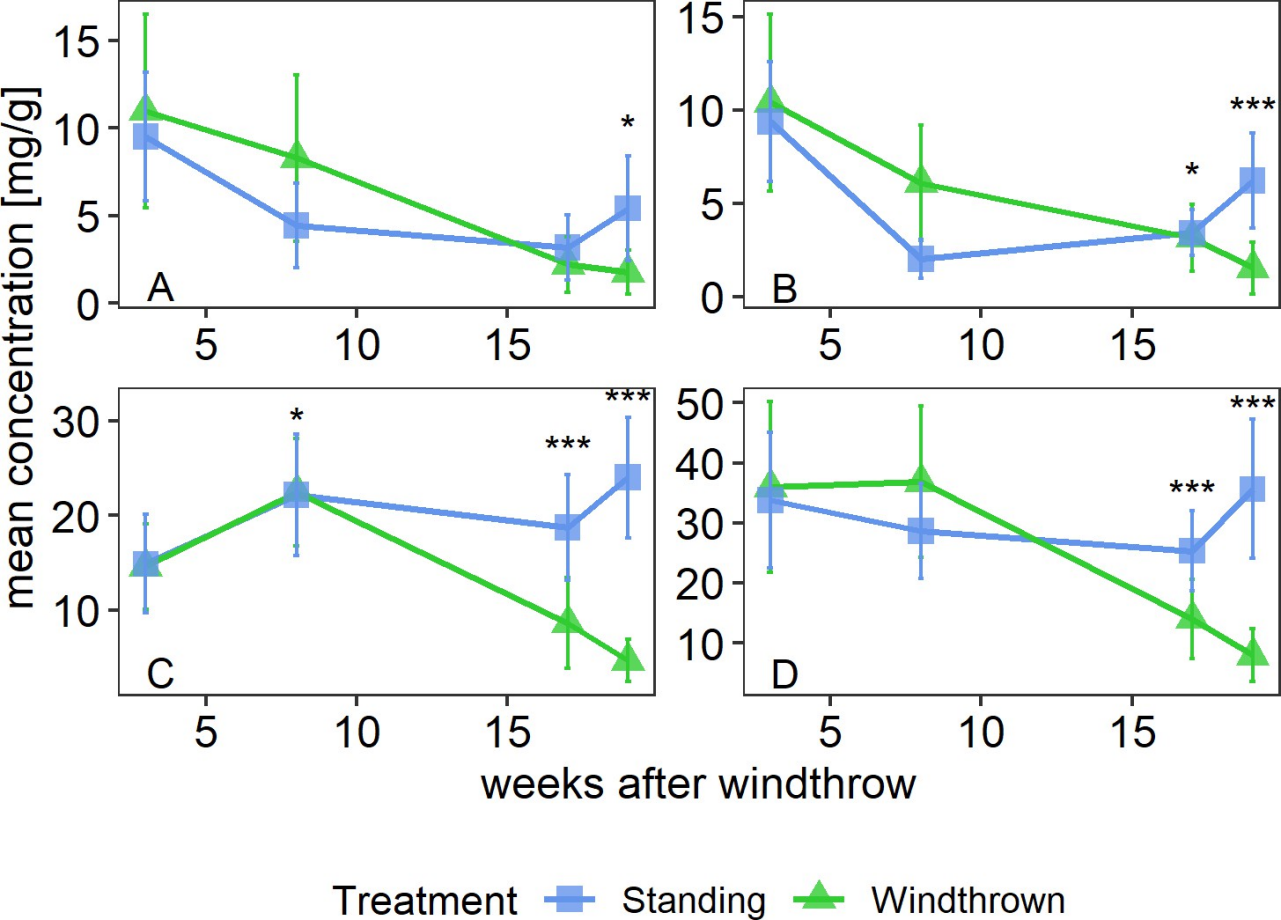
Concentrations (mg/g of dry biomass) of individual soluble sugars: glucose(A), fructose (B) and sucrose(C) and on the sum of individual soluble sugars (D) in Norway spruce trees over a period of several weeks following a windthrow event. Statistical differences between treatments at specific time points are indicted with ***: P<0.001, **: p<0.01, *: p<0.05.

Over the approximately 4-month period of measurement, total soluble sugar concentrations in standing trees increased slightly while concentrations in windthrown trees decreased from first to last sampling day (Fig 2). Over these 4 months, the sucrose concentrations in windthrown trees decreased by 9.9 mg/g (-68.1%), showing a significant difference to standing trees, which had sucrose levels of 14 mg/g (+61.3%) greater (p < 0.001, Wilcoxon rank sum test; S1 Table 1 in S1 File). Differences in treatments for glucose and fructose were not as substantial with windthrown trees experiencing a decrease of 8.8 mg/g (-85.2%) in glucose and 9.1 mg/g (-83.9%) in fructose concentration and standing trees showing a decrease of 4.1 mg/g (-43.1%) for fructose and 3.1 mg/g (-33.7%) decrease for glucose (p < 0.01 and p < 0.05, Wilcoxon rank sum test; S1 Table 1 in S1 File).

**Fig 3:**
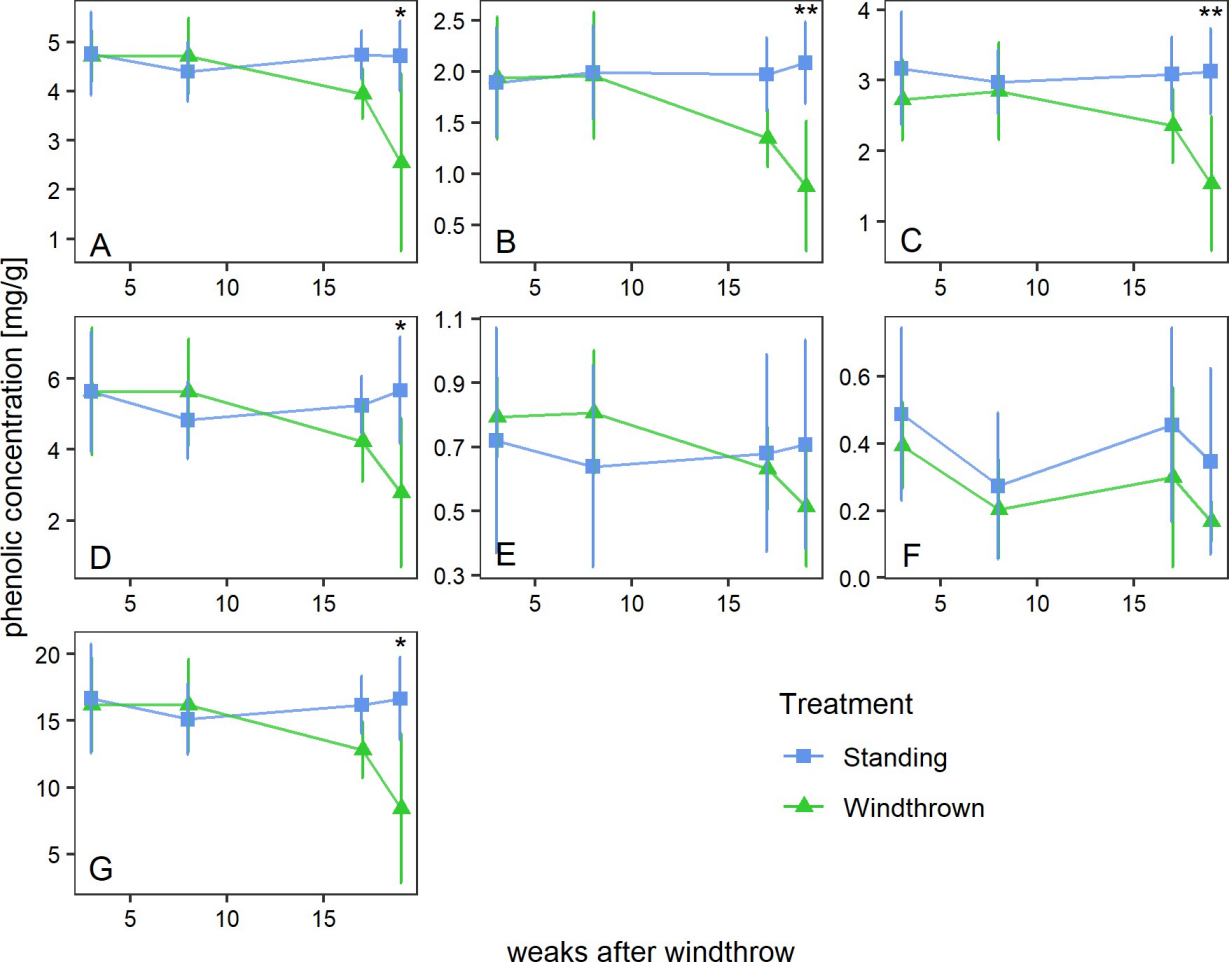
The effect of windthrow on concentrations of phenolic compounds in Norway spruce. A: Astringin; B: Catechin; C: Isorhapontin; D: Proanthocyanidin B1; E: Taxifolin glucoside; F: Taxifolin; G: sum of all phenolic concentrations. Statistical differences between treatments at specific time points are indicted with **: p<0.01, *: p<0.05. Bars depict (+/-) standard deviation for the 4 different time points.

Phenolic substances showed similar trends as soluble sugars (Fig 3 and S1 Table 3 in S1 File). Standing trees showed no noteworthy change of total phenolic concentration during the sampling campaign. Windthrown trees showed a significant 47.9% decrease in total phenolic concentrations after 4 months (p < 0.05, Wilcoxon rank sum test; S1 Table 2 in S1 File). In windthrown trees, all individual compounds experienced a substantial decrease of 35–60% over the 4-month period, especially, catechin (-54.92%) and taxifolin (-58.97%). While most compounds in standing trees showed minor increases, taxifolin (-2.78%) and isorhapentin (-1.27%) showed slightly decreasing concentrations over the sampling period.

**Fig 4:**
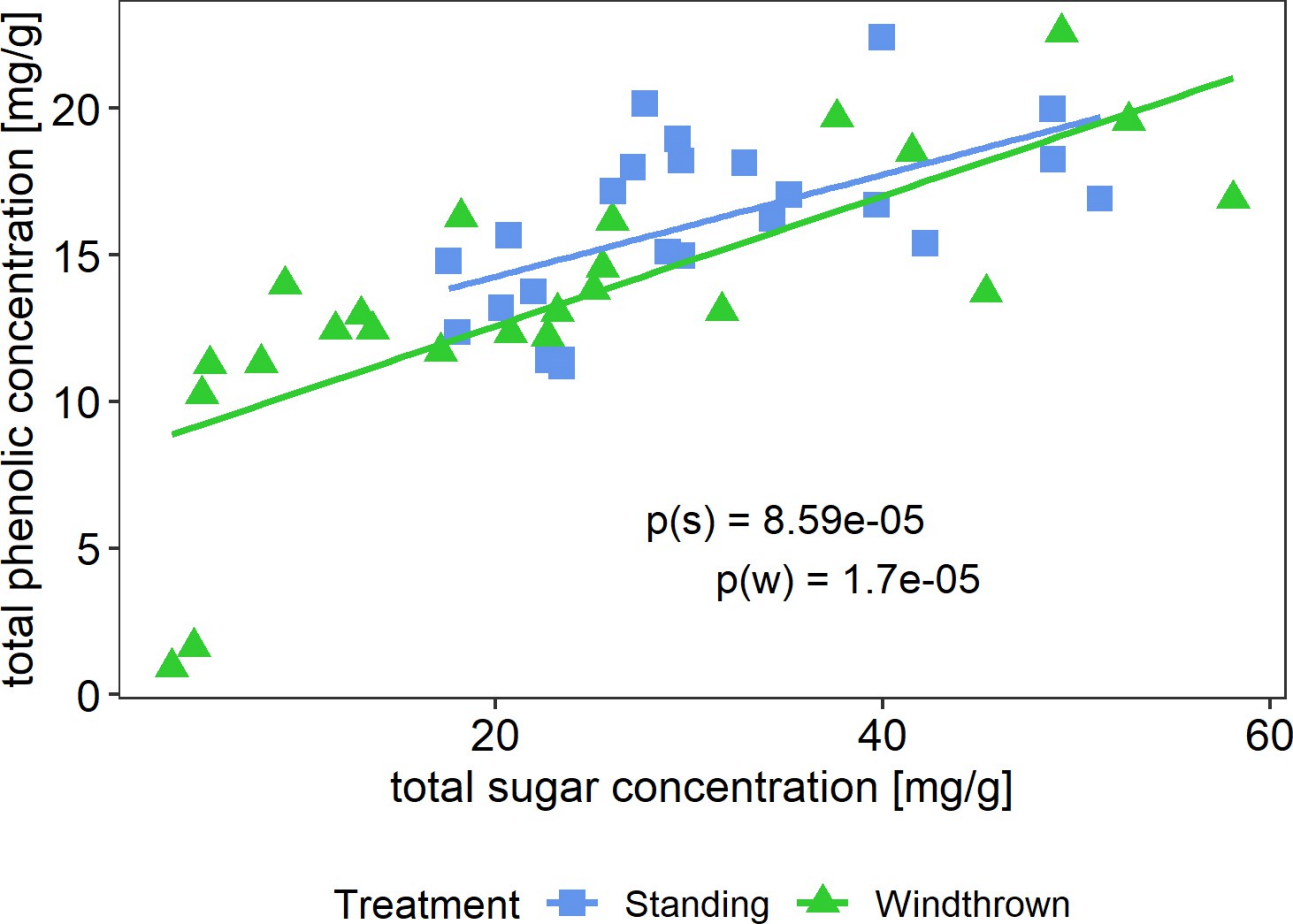
Relationship between sugar and phenolic concentrations of windthrown trees and standing control trees. Dots represent measurements of individual trees. P(s) shows the p-value of standing trees, p(w) of windthrown trees.

Phenolic and soluble sugar concentrations show a distinct positive relationship (Fig 4). Higher sugar concentrations correlate with higher phenolic concentrations in both treatments (p < 0.001, linear regression model; S1 Table 3 in S1 File). Standing trees show a tendency towards higher sugar and phenolic concentration, but the slope shows a similar trend for both treatments.

### Volatile measurements

**Fig 5:**
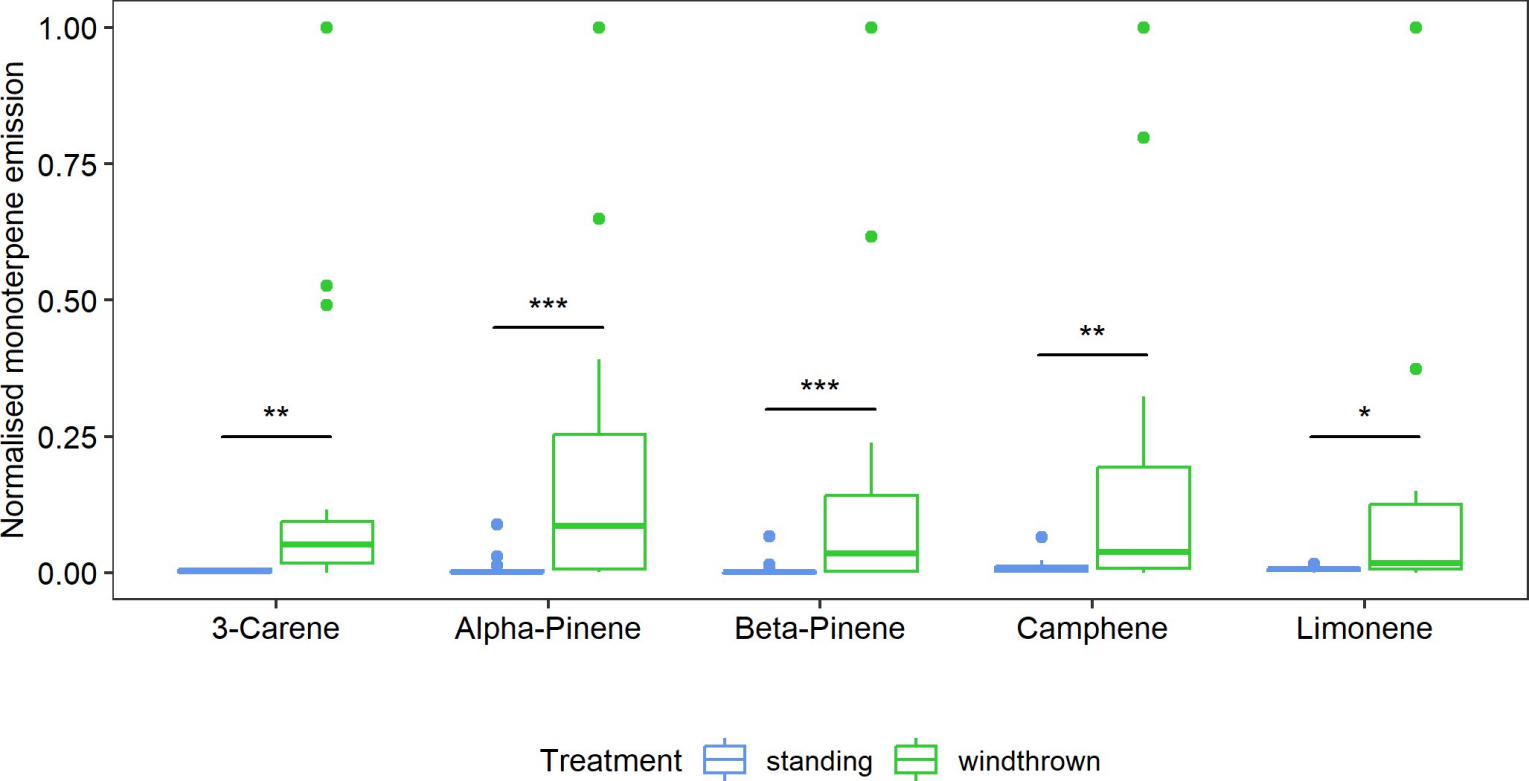
The effect of windthrow on monoterpene emission from Norway spruce trunks as measured with a mobile GC-MS. Data are normalised to highest value within each time series as pooled data. ***: P<0.001, **: p<0.01, *: p<0.05. Normalisation was done using equation shown in ‘Data analysis’.

Monoterpene emissions from the trunks of standing and windthrown trees differed significantly in concentration for all measured compounds. Emissions of windthrown trees were 25 times more than in standing trees (S1 Table 4 in S1 File). Alpha-pinene is emitted at highest rates in both treatments in relation the other compounds (61.3% in standing, 58.3% in windthrown), followed by beta-pinene and camphene. The fourth most abundant compound was limonene in standing and 3-carene in windthrown trees. With 3-carene showing roughly 90 times greater concentrations in windthrown versus standing trees (p < 0.01 Wilcoxon rank sum test; S1 Table 5 in S1 File), this monoterpene showed the greatest difference. Only limonene did not show such a large significant trend (p < 0.05, Wilcoxon rank sum test; S1 Table 5 in S1 File), although emissions were still higher in windthrown trees (Fig 5).

When investigating the proportions of compounds in windthrown and standing trees, windthrown trees showed a higher emission of 3-carene and beta-pinene in relation to the other substances with 4.2% and 24.1%, respectively, in comparison to about 1.1% and 21.7% in standing trees (S1 Table 4 in S1 File). However, standing trees showed higher proportions of alpha-pinene and camphene with 61.3% and 14.0%, while the share of this substance in windthrown trees was only 58.1% and 11.4%. Trees of both treatments showed similar proportions of limonene with 1.8% in standing and 1.9% in windthrown trees.

## Discussion

Our study shows that windthrown trees underwent a notable decline in vigour and defence capacity, indicated by a decrease of sugars and phenolic concentrations, in contrast to standing and undisturbed trees. This alteration in tree physiology was associated with quantitative changes in concentrations of monoterpenes emissions, which may provide cues to bark beetles for identifying suitable host trees.

### Windthrown trees are less well-defended than standing trees

A reduction in water availability by the uprooting of windthrown trees can reduce photosynthetic activity and therefore lead to a decreased supply of carbohydrates. NSCs are a main substrate for many processes in trees, such as growth, development and the synthesis of defence compounds [30]. Water limitation can limit the production of new carbohydrates and make trees rely on stored NSCs, which can quickly be depleted [31, 32]. Reduced photosynthesis and reduced NSC production caused by windthrow or drought [33] may in turn constrain the production of secondary defence metabolites, and make trees more vulnerable to damaging insects or diseases [34, 35].

Phenolics, as a major group of defence compounds in spruce [36] are stored in different organs of the tree, including the bark and contribute to constitutive and induced defences against insects, microbes and herbivores [28, 37]. Our results indicate that windthrown trees show a decrease of certain flavan-3-ols and stilbenes which are biosynthesised in the phenylpropanoid pathway [38, 39]. Stilbene (astringin and isorhapontin measured here) and flavonoid biosynthesis requires *para*-coumaroyl CoA which is made from phenylalanine, an amino-acid that is involved in the production of most phenolic compounds [36]. Phenylalanine in turn is derived via the shikimate pathway from phosphoenolpyruvate (PEP) and erythrose 4-phosphate, two intermediates of plant central metabolism derived from fixation of carbon or breakdown of glucose [40]. A decreased supply of NSCs can therefore have a direct effect on the ability of trees to produce defence compounds. Several studies have shown similar results in which water stress, e.g. caused by drought can constrict the production of secondary metabolites and therefore decrease the defence capability of spruce trees [9, 28, 41].

### Monoterpene emission increase in windthrown trees

The large increase in monoterpene emissions of windthrown spruce trees observed in our study has been reported by others also. Trees cut down and left in the field showed higher total monoterpene emission than control trees left standing [18]. A previous study on artificially uprooted trees showed a large drop in phloem monoterpene concentration over several months, which is also consistent with a high rate of volatilization [42]. Because spruce monoterpenes are typically stored in resin ducts [43], increased emission might occur if resin ducts had been broken during windthrow. Although the bark of all trees measured in our study was intact, with no cracks of leaking resin visible, the death of cells on windthrow and associated disintegration of membranes may lead to increased emission without any obvious damage.

The increased emission from windthrown trees could also arise from the release of newly synthesized monoterpenes not associated with resin ducts or other storage structures. Various abiotic factors, such as increased light, increased temperature and moderate drought have been reported to lead to elevated monoterpene biosynthesis and emission in conifers and other woody plants [41, 44], and the uprooting caused by windthrow leads to drought effects [42]. The elevated emission we measured is a typical conifer response not only to abiotic stresses, but also to herbivory or pathogen infection [45]. Extensive studies on Norway spruce have demonstrated that herbivore damage (or damage simulated by mechanical wounding or methyl jasmonate treatment) leads to increases in monoterpene emission rates [46, 47]. Even artificially uprooted trees respond to methyl jasmonate treatment with an increased content of phloem monoterpenes, which could lead to elevated emission rates [42]. Such an increase could represent a direct defence response to biotic attackers since volatile monoterpenes are toxic and repellent to many insect herbivores and microbial pathogens [48]. Partially-fallen trees that remain alive could benefit from increased defence if they are at greater risk of further attack.

Spruce monoterpenes are formed from the intermediate geranyl diphosphate, which is in turn made from products of the methylerythritol phosphate pathway [49]. Precursors of the methylerythritol phosphate pathway may be derived directly from photosynthetic carbon fixation or from NSCs via glycolysis in non-photosynthetic cells. The reduced NSC concentrations present in windthrown trees are inconsistent with an increase in monoterpene formation, which may indicate that monoterpene biosynthesis is not limited by NSC substrate supply or that the increased emission is not a consequence of increased biosynthesis, but rather due to the increased permeability of storage structures. Additional research is needed to understand the mechanism for elevated monoterpene emission from windthrown spruce trees.

The monoterpenes of Norway spruce are emitted as a complex blend. Studies have shown alpha-pinene, beta-pinene and limonene to be the most abundant monoterpenes to be emitted from Norway spruce [17, 50]. Our results are in general agreement, and we discovered highest differences between standing and windthrown trees for alpha-pinene, 3-carene and beta-pinene. When examining the relative proportions of the blend, windthrown trees showed a higher relative emission of 3-carene and beta-pinene while standing trees had higher relative emissions of alpha-pinene and camphene in relation to the other substances. Additionally, oxygenated monoterpenes may play important roles in primary attraction [18, 51] but were not measured in this study.

### Changes in monoterpene emissions as a potential cue for bark beetles to identify physiologically stressed host trees

A strong link between physiological stress in trees and infestations by bark beetle has also been noted by earlier works [52, 53]. The depletion of NSCs we observed and the reduction in phenolic accumulation could facilitate a successful infestation by bark beetles and their associated fungi, as the tree’s defence capacity declines [34, 54]. Trees from our experiment showed a significant decrease in phenolic defences after four to five months, although depending on the extent of windthrow this could take longer for individual trees [42].

Bark beetles have been shown to locate host tree species and avoid non-hosts based on their vastly different olfactory cues [17, 21, 55]. However, it has not been found out if *Ips typographus* can perceive differences in volatile emissions between stressed and unstressed host trees and therefore discriminate between more suitable hosts with lowered defences and less suitable hosts with greater defences [16, 18, 56]. Rather than responding to differences in the total emissions of monoterpenes, it has been suggested that beetles could react to qualitative changes in the emission profiles of trees [18]. For example, 3-carene, which is emitted in higher proportions from windthrown trees could act as an attractant to such trees. However, monoterpenes have shown very differing effects on beetles depending on the emitted concentrations, so they can be both attracting or repelling depending on the concentration [57]. This way, beetles could distinguish potential host trees that are under stress and poorly defended based on strongly increasing emissions of certain terpenes, but more studies are needed to prove this phenomenon.

## Conclusion and outlook

The results of this study show the effect of physiological stress on tree vitality, defence and monoterpene emission after windthrow disturbance. Our work shows implications regarding monoterpene stress markers and their effect on bark beetle behaviour. An increased monoterpene concentrations and changes in emission profiles could act as indicator for beetles to select appropriate host trees. Yet, more investigations on differences in amount and proportions of certain substances and their effect on beetle behaviour could shed light onto the effects of climate change on tree physiology including tree-beetle-interactions. Understanding the effect of host tree volatiles on primary attraction could help optimizing beetle traps for early-swarming periods. Using highly specialised baits to capture pioneer beetles could be more effective in reducing beetle population growth and allow hampering and delaying outbreaks. Given the severity of recent *I*. *typographus* outbreak levels in Central Europe, each and every measure to slow bark beetle populations will be important to buy time for other forest management actions against outbreak. Our results further underline the importance of removing windthrown trees from forests as soon as possible to prevent a beetle population build-up that originates from these less resistant and potentially more attractive trees to bark beetle attacks.

## Supporting information

S1 File Statistical analysis for soluble sugars, phenols and monoterpenes of standing and windthrown trees. (DOCX)

S2 File Non-structural carbohydrate and phenolic concentrations as well as monoterpene emissions from standing and windthrown spruce trees. (XLSX)

S1 Fig Overlay of raw exemplary mass spectra from monoterpene emission measurements from standing and windthrown spruce trees. The most important peaks are labelled.
(TIF)

S2 Fig Raw exemplary mass spectra from monoterpene emission measurement from tree 3 (windthrown) measured on 03.05.2022. The most important peaks are labelled.
(TIF)

S3 Fig Raw exemplary mass spectra from monoterpene emission measurement from tree 1 (windthrown) measured on 11.05.2022. The most important peaks are labelled.
(TIF)

S4 Fig Raw exemplary mass spectra from monoterpene emission measurement from tree 10 (standing) measured on 23.05.2022. The most important peaks are labelled.
(TIF)

S5 Fig Raw exemplary mass spectra from monoterpene emission measurement from tree 11 (standing) measured on 23.05.2022. The most important peaks are labelled.
(TIF)

S6 Fig Raw exemplary mass spectra from monoterpene emission measurement from tree 4 (windthrown) measured on 31.05.2022. The most important peaks are labelled.
(TIF)

S7 Fig Raw exemplary mass spectra from monoterpene emission measurement from tree 8 (standing measured on 31.05.2022). The most important peaks are labelled.
(TIF)
