# Supplementary material for: Windthrow causes declines in carbohydrate and phenolic concentrations and increased monoterpene emission in Norway spruce
Source: PLoS One. 2024 May 28;19(5):e0302714. doi: 10.1371/journal.pone.0302714 (PMC11132463; doi:10.1371/journal.pone.0302714)
Supplement: S1 File — (DOCX) [file pone.0302714.s001.docx]

Lehmanski et al. Supplementary data

**S1 Table 1:** Statistical analysis for soluble sugars of standing and windthrown trees over a 4-month period. Shapiro-Wilk to test for normal distribution; Wilcoxon rank sum test for non-parametric statistical comparison of windthrown and standing trees. Sum of individual compounds is displayed as Total.

|  | Total | Glucose | Fructose | Sucrose |
| --- | --- | --- | --- | --- |
| Shapiro Wilk | 0.3231 |  | | |
| Wilcoxon rank sum test between treatments | | | | |
| Overall | 0.07285 | 0.9716 | 0.8957 | 0.001143 |
| 09.03.2022 | 0.2041 | 0.6712 | 0.6005 | 0.9227 |
| 13.04.2022 | 0.7724 | 0.01197 | 0.1082 | 0.001143 |
| 14.06.2022 | 0.01408 | 0.7768 | 0.3493 | 0.007393 |
| 29.06.2022 | 0.0002778 | 0.002821 | 0.02108 | 3.629e-05 |

**S1 Table 2:** Statistical analysis of dependency of sugars and phenols of standing and windthrown trees. Relationship between dependent variables sugar and phenols is evaluated with a linear regression model.

|  | Total | Astringin | Catechin | Isorhapontin | Proanthocyanidin B1 | Taxifolin glucoside | Taxifolin |
| --- | --- | --- | --- | --- | --- | --- | --- |
| Shapiro-Wilk | 0.001761 | | | | | | |
| Overall | 0.01937 | 0.1832 | 0.007119 | 0.0004377 | 0.3533 | 0.8205 | 0.07447 |
| 09.03.2022 | 0.4848 | 1 | 0.6991 | 0.09307 | 0.8182 | 0.5887 | 0.3095 |
| 13.04.2022 | 0.8182 | 0.5887 | 0.4704 | 0.3939 | 0.4704 | 0.3939 | 0.2403 |
| 14.06.2022 | 0.09307 | 0.3095 | 0.05424 | 0.1488 | 0.6884 | 0.4848 | 0.132 |
| 29.06.2022 | 0.02597 | 0.02472 | 0.002165 | 0.004329 | 0.04113 | 0.229 | 0.2403 |

**S1 Table 3:** Statistical analysis of dependency of sugars and phenols of standing and windthrown trees. Relationship between dependent variables sugar and phenols is evaluated with a linear regression model.

| Treatment | p-value associated with t-value |
| --- | --- |
| Standing | 8.59e-05 |
| Windthrown | 1.7e-05 |

**S1 Table 4:** Mean monoterpene concentrations of standing and windthrown trees, relative emissions and relation between both treatments. Total number of measurements: 25. Sum of individual compounds is displayed as Total.

| Compound | Standing trees [ppm] | % of total emissions | Windthrown trees [ppm] | % of total emissions | Times greater concentrations in windthrown than standing trees |
| --- | --- | --- | --- | --- | --- |
| 3-Carene | 0.01089906 | 1.14% | 0.947367 | 4.23% | 90 |
| Alpha-Pinene | 0.582401 | 61.31% | 13.05652 | 58.31% | 23 |
| Beta- Pinene | 0.2059339 | 21.68% | 5.400564 | 24.12% | 27 |
| Camphene | 0.1337029 | 14.07% | 2.566915 | 11.46% | 19 |
| D-Limonene | 0.0169064 | 1.77% | 0.4187544 | 1.87% | 24 |
| Total | 0.9498432 |  | 23.33996 |  | 25 |

**S1 Table 5:** Statistical analysis for monoterpene emissions of standing and windthrown trees: Shapiro-Wilk to test for normal distribution; Mann-Whitney-U test for comparing sample means of individual compounds; Kolmogorov-Smirnov to assess continuous distribution.

|  | total | 3-Carene | Alpha-Pinene | Beta-Pinene | Camphene | Limonene |
| --- | --- | --- | --- | --- | --- | --- |
| Shapiro Wilk | 2.2e-16 |  |  |  |  |  |
| Wench Anova | 0.0003094 |  |  |  |  |  |
| Mann-Whitney-U |  | 0.001365 | 0.0001311 | 0.00034 | 0.005032 | 0.01414 |
| Kolmogorov |  | 0.001781 | 0.0008856 | 0.0006164 | 0.01954 | 0.02075 |
