# Supplementary figures and images for: Windthrow causes declines in carbohydrate and phenolic concentrations and increased monoterpene emission in Norway spruce
Source: PLoS One. 2024 May 28;19(5):e0302714. doi: 10.1371/journal.pone.0302714 (PMC11132463; doi:10.1371/journal.pone.0302714)

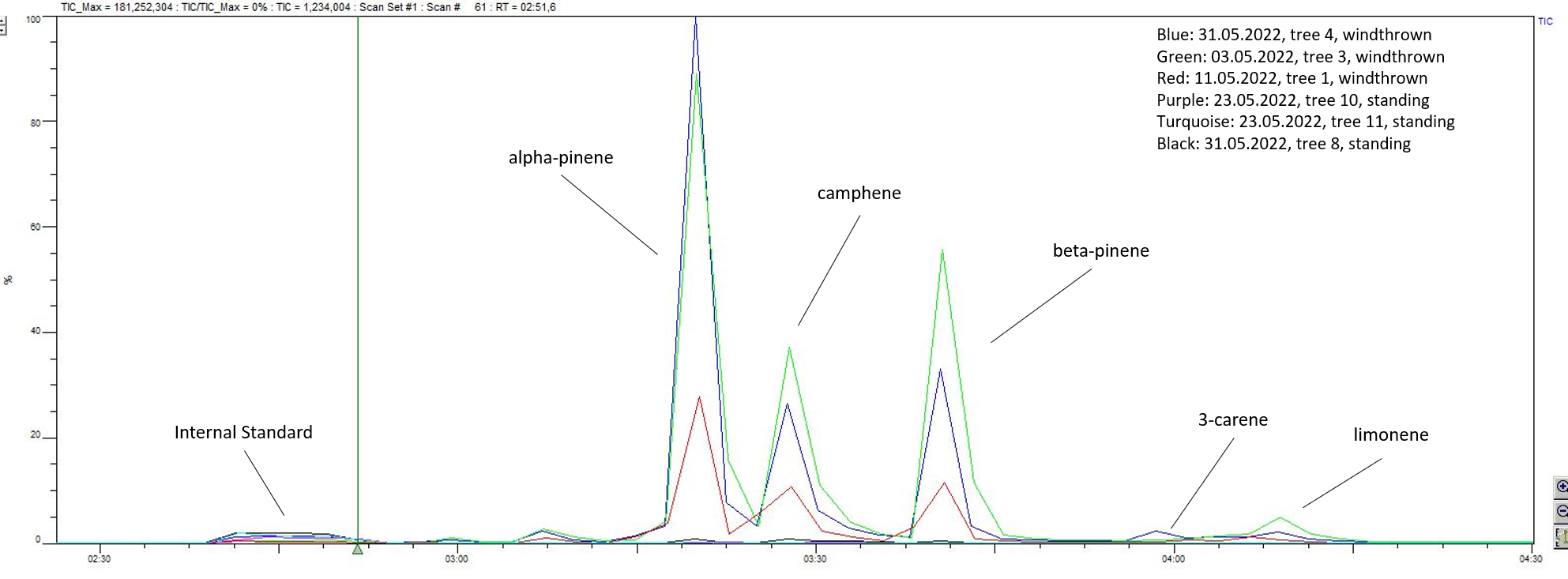

Supplement: S1 Fig — The most important peaks are labelled. (TIF) [file pone.0302714.s003.tif]

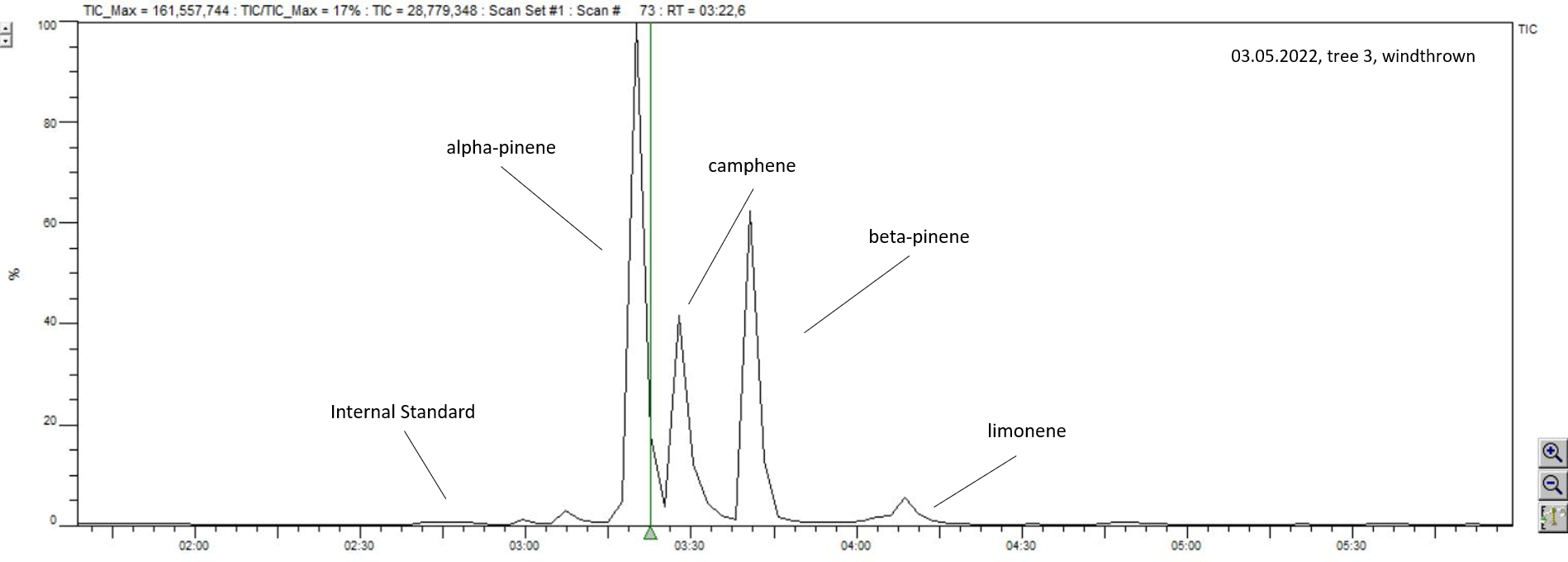

Supplement: S2 Fig — The most important peaks are labelled. (TIF) [file pone.0302714.s004.tif]

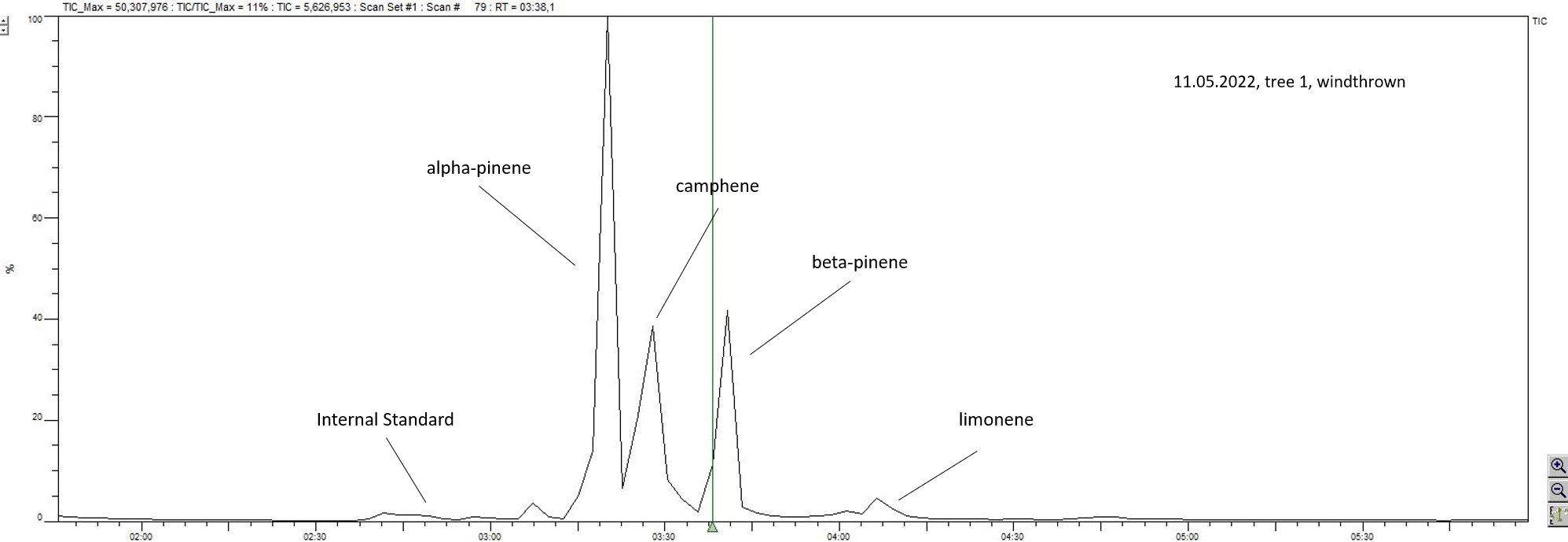

Supplement: S3 Fig — The most important peaks are labelled. (TIF) [file pone.0302714.s005.tif]

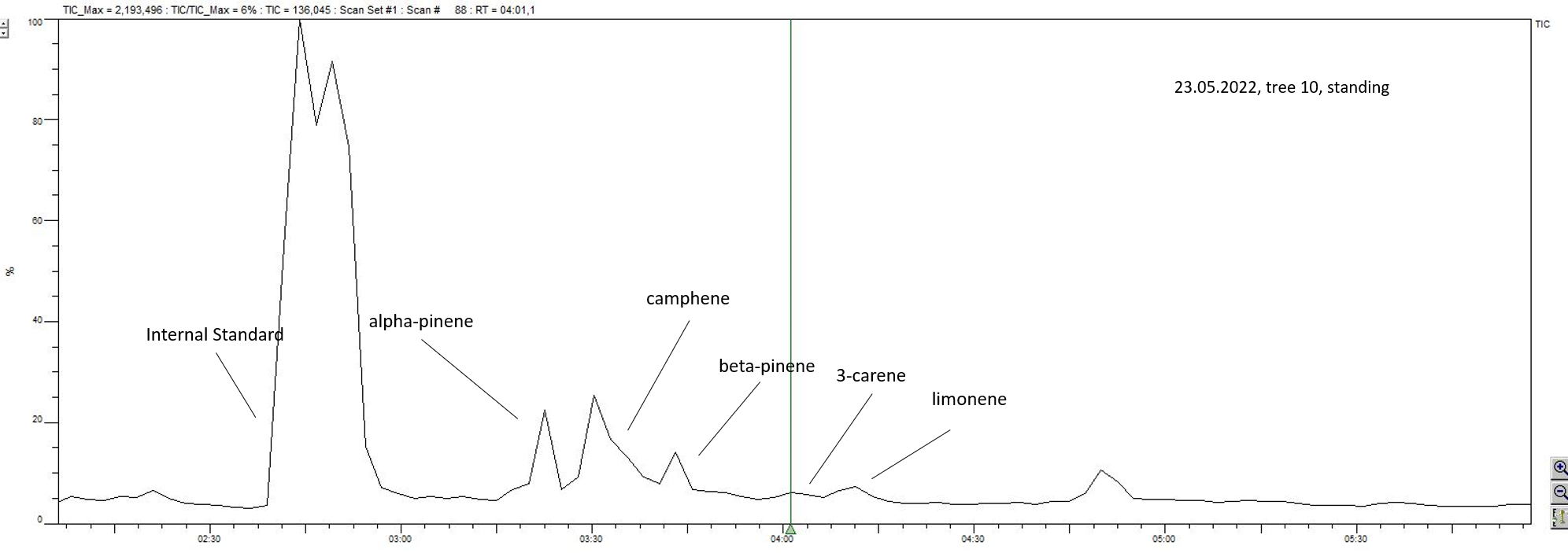

Supplement: S4 Fig — The most important peaks are labelled. (TIF) [file pone.0302714.s006.tif]

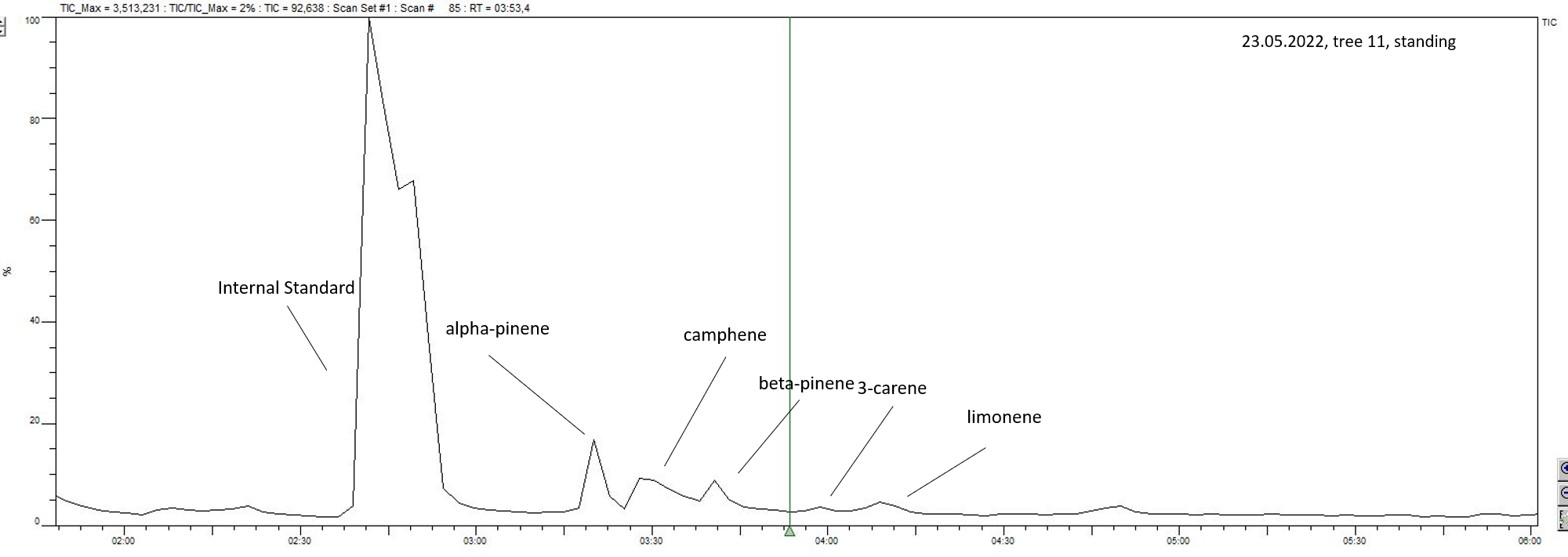

Supplement: S5 Fig — The most important peaks are labelled. (TIF) [file pone.0302714.s007.tif]

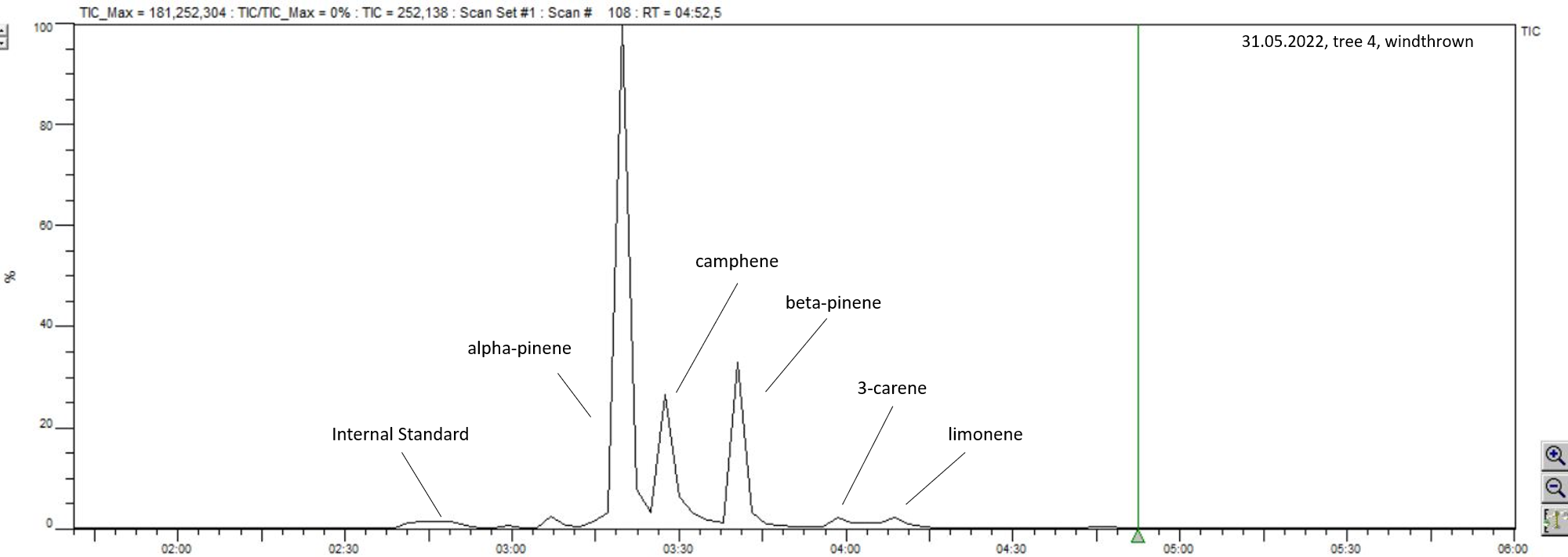

Supplement: S6 Fig — The most important peaks are labelled. (TIF) [file pone.0302714.s008.tif]

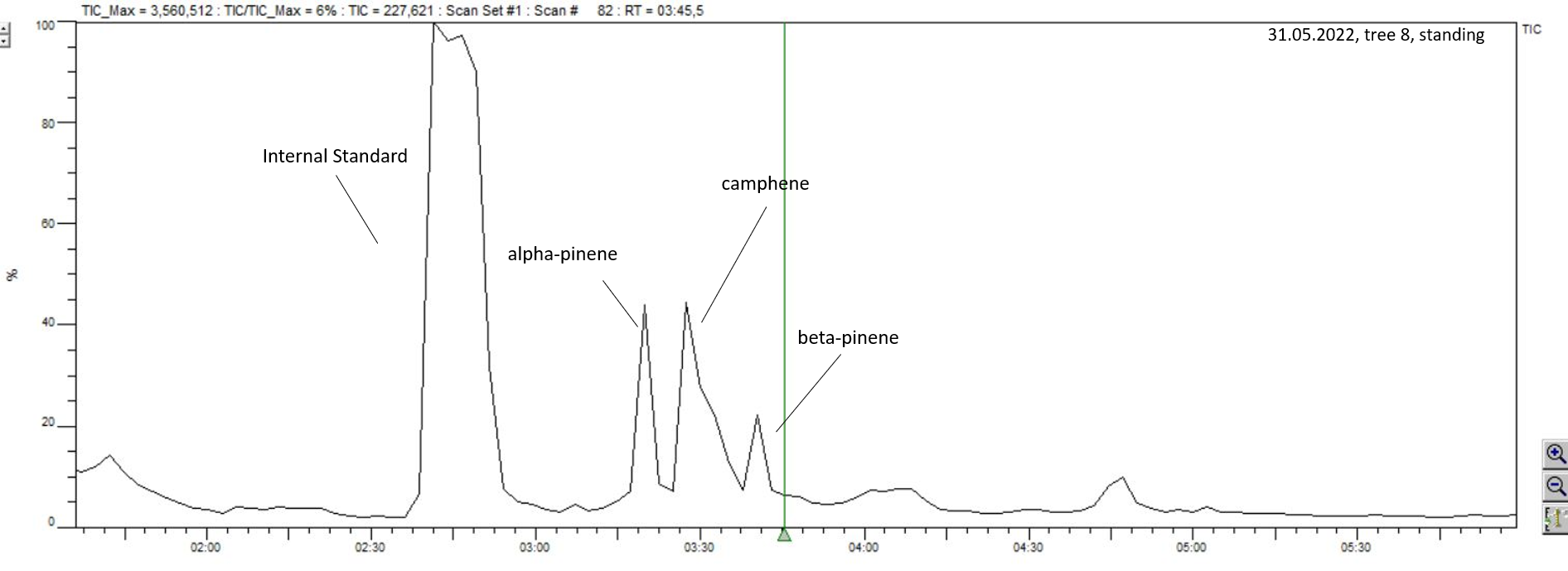

Supplement: S7 Fig — The most important peaks are labelled. (TIF) [file pone.0302714.s009.tif]
